# How does multiannual plastic mulching in strawberry cultivation influence soil fungi and mycotoxin occurrence in soil?

**DOI:** 10.1007/s12550-022-00451-5

**Published:** 2022-03-22

**Authors:** Maximilian Meyer, Gabriele Ellen Schaumann, Katherine Muñoz

**Affiliations:** 1 iES Landau, Institute for Environmental Sciences, Group of Environmental and Soil Chemistry, University Koblenz-Landau, Landau, Germany; 2 iES Landau, Institute for Environmental Sciences, Group of Organic and Ecological Chemistry, University Koblenz-Landau, Landau, Germany

**Keywords:** Plastic mulching, Soil fungi, Ergosterol, Deoxynivalenol, Nivalenol, Zearalenone

## Abstract

The production of mycotoxins is often interpreted as fungal response to cope with unfavorable growth conditions induced by toxic substances, environmental and biological factors. Soil covers influence soil environment, which consequently can change the abundance and composition of microbial communities. We investigated how plastic coverage (PC) influence soil fungi and mycotoxin occurrence (deoxynivalenol, nivalenol and zearalenone) compared to the traditional straw coverage (SC) in dependence of soil depth and time in a 3-year field experiment in strawberry cultivation. In total, 300 soil samples, resulting from two treatments, three soil layers, and ten sampling dates (*n* = 5), were analyzed for mycotoxins and ergosterol (proxy for soil fungal biomass) with liquid chromatography high resolution mass spectrometry and high-performance liquid chromatography with UV-detection, respectively. The modified microclimate under PC had no significant influence on fungal biomass, whereas SC promoted fungal biomass in the topsoil due to C-input. Mycotoxins were detected under both cover types in concentrations between 0.3 and 21.8 µg kg^−1^, mainly during strawberry establishment period and after fungicide application. Deoxynivalenol had the highest detection frequency with 26.3% (nivalenol: 8.3%, zearalenone: 8.7%). This study confirmed the in situ production of mycotoxins in soil, which seems mainly triggered by field treatment (fungicide application) and plant growth stage (establishment period) rather than on mulching type. Further investigations are necessary to better understand the influence of different agricultural practices and soil types on the production and fate of mycotoxins.

**Supplementary information:**

The online version contains supplementary material available at 10.1007/s12550-022-00451-5.

## Introduction

Mycotoxins are secondary metabolites produced by several fungal species and can exert a wide array of toxic effects to humans and animals (Vanhoutte et al. 2016). The frequent mycotoxin contamination of food and feed commodities, exceeding the maximum limits set in many countries to reduce mycotoxin exposure, can result in large economical losses (Robens and Cardwell 2003; Wilson et al. 2018). Mycotoxins are primarily produced by the genera *Aspergillus* spp., *Penicillium* spp., and *Fusarium* spp. (Sweeney and Dobson 1998). *Aspergillus* spp. and *Penicillium* spp. infest food and feed mainly during storage, whereas the *Fusarium* spp. are field fungi, which can grow in soils and infest various food crops during different growth stages (Elmholt 2008). Deoxynivalenol (DON), nivalenol (NIV), and zearalenone (ZEN) are frequently produced mycotoxins by *Fusarium* spp. in temperate climates (Table 1) (Elmholt 2008). Although the role of mycotoxins in the fungal life cycle is not yet completely understood, mycotoxin production of *Fusarium* spp. is often seen as stress response to unfavorable growth conditions (Reverberi et al. 2010), including fungicides, high temperatures, water and nutrient scarcity, and competition (Magan et al. 2002; Schmidt-Heydt et al. 2008; Reverberi et al. 2010; Venkatesh and Keller 2019).

**Table 1:**
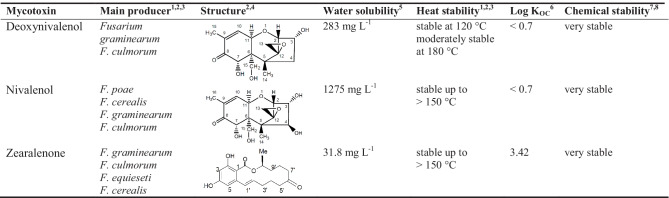
Main producer, synthesis conditions and physicochemical properties of relevant mycotoxins in temperate climates

^1^EFSA (2004a)

^2^EFSA (2013)

^3^EFSA (2011)

^4^EFSA (2004b)

^5^USEPA (2012)

^6^Schenzel et al. (2012a)

^7^Lauren and Smith (2001)

^8^Pitt et al. (2012)

Plastic mulching has become a widely applied agricultural practice to improve growth and harvest conditions of many food crops, because it increases soil temperature and moisture, suppresses weed growth, and reduce nutrient (fertilizer) leaching by an impeded rainfall infiltration (reviewed by Steinmetz et al. 2016). Elevated soil temperature and moisture can enhance fungal growth (Dighton 2003; Pietikåinen et al. 2005; Bárcenas-Moreno et al. 2009) and shift microbial community (Castro et al. 2010), which might stress soil fungi due to competition and nutrient scarcity (Reverberi et al. 2010; Venkatesh and Keller 2019). This assumption is emphasized by decreasing soil organic matter (SOM) under plastic mulching (reviewed by Steinmetz et al. 2016), which can result in a limited substrate for fungal growth and hence stronger competition for nutrients (Reverberi et al. 2010; Swer et al. 2011). The high soil temperatures under plastic mulching in the summer season can exceed the growth optima and even the growth maxima of *Fusarium* spp. (Sweeney and Dobson 1998), which might additionally stress soil fungi (Schmidt-Heydt et al. 2008; Muñoz et al. 2015). The impeded rainfall infiltration under plastic mulching decrease leaching (Subrahmaniyan et al. 2006) but might lead to an accumulation of mycotoxins in surface soil, which can become susceptible to leaching after plastic mulch removal. First indications that plastic mulching can affect mycotoxin occurrence and shift fungal community toward mycotoxigenic fungi were reported by Muñoz et al. (2015, 2017). But it is largely unknown how mycotoxin levels and fungal biomass can change during the multiannual use of plastic mulching due to its effects on the soil environment.

However, this is an important information, as studies by Schenzel et al. (2012b) and by Kolpin et al. (2014) showed that DON, NIV, and ZEN have the potential to contaminate running and groundwaters due to runoff and leaching from contaminated fields. Especially, DON and NIV have a high water solubility (Table 1) and can thus remain in the soil solution and possibly undergo plant uptake (Rolli et al. 2018) or microbial degradation (Vanhoutte et al. 2016). In contrast to the well-documented mycotoxin occurrence in food and feed (Elmholt 2008), the available literature is very scarce about the occurrence, production, and fate of mycotoxins in agricultural soil and how this is linked to agricultural practices.

The objective of this study was to investigate the influence of plastic coverage (PC) on soil fungi and occurrence of the mycotoxins DON, NIV, and ZEN in soil compared to the traditional straw coverage (SC) in dependence of soil depth and time in a 3-year field experiment in strawberry cultivation. We hypothesize that (1) elevated soil temperature and moisture under PC compared to SC promote fungal growth and expand fungal community, and (2) the adaption of the soil fungi to the modified microclimate under PC will trigger a higher mycotoxin occurrence under PC compared to SC.

## Material and methods

This study was part of a 3-year field experiment on the influence of plastic mulching on various soil parameters and biogeochemical soil processes, investigated on the example of a commercial strawberry cultivation (Meyer et al. 2020, 2021a, b). This paper repeats the relevant information to retrace experiment and methods and shows new data about mycotoxins (sampling T0–T2 and T7–T9), ergosterol (sampling T0–T2 and T7–T9), MBC:SOC ratios (sampling T7–T9), and MBN:TN ratios (sampling T0–T9).

### Site description and field establishment

The study was conducted on a commercial strawberry field in southwestern Germany (49°11′N, 8°10′E, 130 m a.s.l.) from 2016 to 2019. The soil was a silt loam (Anthrosol), according to FAO soil classification (IUSS Working Group WRB 2015), with a texture of 7 ± 2% sand, 83 ± 5% silt, and 10 ± 3% clay in the 0–60 cm soil layer. A ridge-furrow system was established in the field in late-June 2016 with subsurface drip irrigation and black plastic-covered ridges (polyethylene, 50 µm). Strawberry seedlings (*Fragaria* × *ananassa*, “Malwina”) were planted in double rows at the ridges in mid-July 2016. The initially bare furrows were yearly covered with wheat straw, starting in April 2017.

### Experimental design and soil sampling

A semi-controlled field experiment with a homogeneous soil type was used, which represents current agricultural practice and avoids masking of treatment effects by landscape variation and edge effects. One treatment area with plastic-covered (PC) and one with straw-covered ridges (SC) were chosen (both 21 × 10 m), in which respectively five sampling plots (10 × 1.5 m) were randomly chosen for soil sampling: PC (*n* = 5) and SC (*n* = 5). In the SC treatment area, plastic coverage was manually removed after field setup in July 2016 and the bare soil ridges were yearly covered with wheat straw, starting in April 2017. The agricultural practices (irrigation, fertilization and pesticide application) were identical in both treatments during sampling period. In brief, subsurface drip irrigation was applied yearly from March until September depending on weather conditions and fertilization (15 kg N, 5 kg P, 30 kg K, 2 kg Mg) was weekly conducted via drip irrigation during an 8-week period from March to May each year. Fungicides (1 kg ha^−1^ Switch and 2 kg ha^−1^ Teldor) were applied yearly during bloom of strawberries.

Ten soil samplings were conducted during a 3-year period of strawberry cultivation: during the establishment period of strawberry plants, three samplings in 2-month intervals were conducted after planting from late-July to late-November in 2016 (T0–T2) to identify a potential short-term impact of PC on soil parameters and processes after field setup and strawberry plantation (Meyer et al. 2020). In order to investigate the influence of PC on soil parameters and processes during multiannual application in strawberry cultivation, five further samplings were conducted on 25 April 2017 (T3), 9 October 2017 (T6), 3 May 2018 (T7), 11 October 2018 (T8), and 23 July 2019 (T9) (Meyer et al. 2021b). Additionally, two further samplings were conducted on 19 June (T4) and 18 July (T5) in 2017 after fungicide application to estimate the influence of both coverage types (PC and SC) on fungicide residues in soil and their impact on mycotoxin occurrence, microbial biomass and SOM decomposition (Meyer et al. 2021a).

Composite soil samples (five single cores) were taken in the ridges of each sampling plot in the surface, root and subsoil layer (0–10, 10–30, and 30–60 cm) with stainless steel soil sampling rings (0–10 cm) and a boring rod (> 10 cm).

### General soil parameters

Soil organic carbon (SOC) and C:N ratio can influence fungal growth (Bossuyt et al. 2001; Swer et al. 2011) as well as soil temperature, moisture and pH can influence both fungal growth and mycotoxin biosynthesis (Marin et al. 1995; Sweeney and Dobson 1998; Ramirez et al. 2006; Schmidt-Heydt et al. 2008). For this reason, the temperature and moisture data of the 3-year field study published in Meyer et al. (2021b) are presented in the results. Soil temperature and moisture were measured at the 5, 15, and 35 cm soil depth under PC and SC with a field measuring station (ecoTech®, Bonn, Germany), while the air temperature and precipitation data were received from the weather station Landau-Wollmesheim (Agrarmeteorologie, Rheinland-Pfalz). The data of soil pH (0.01 M CaCl_2_), SOC and C:N ratio, published in Meyer et al. (2020, 2021a, b), was summarized in respectively one figure and added to the supplementary information (SI Figs. 1–3).

**Fig. 1:**
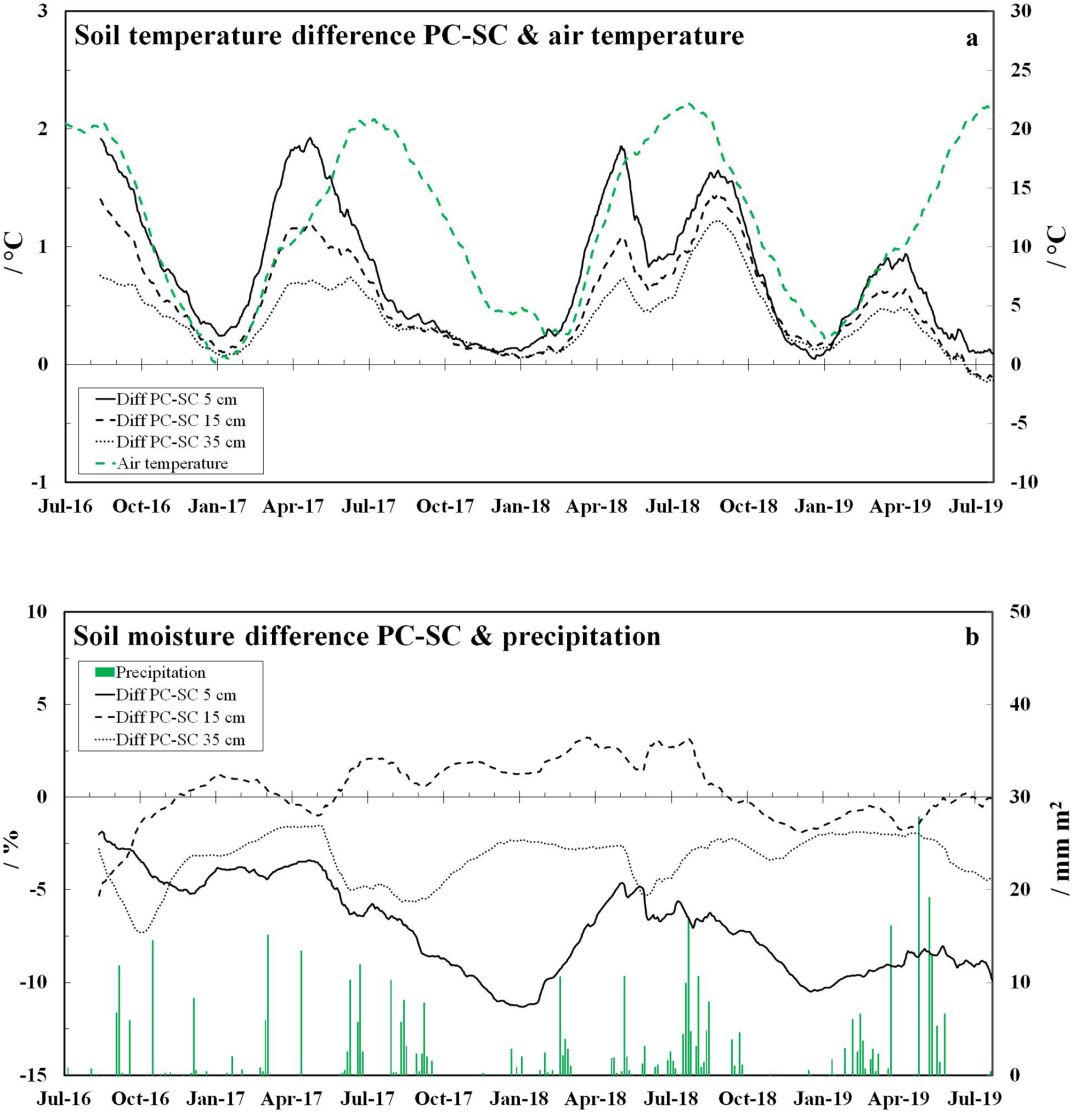
Soil temperature and moisture within the 3-year field experiment. **a** Smoothen soil temperature differences, based on daily means, between plastic coverage (PC) and straw coverage (SC) measured at 5, 15, and 35 cm soil depth and daily mean air temperature measured 2 m above ground. **b** Smoothen soil moisture differences, based on daily means, between plastic coverage (PC) and straw coverage (SC) measured at 5, 15, and 35 cm soil depth and daily precipitation (published in Meyer et al. (2021b))

### Analysis of microbial soil parameters

The microbial biomass carbon (MBC) to nitrogen (MBN) ratio, the ratios of MBC and MBN to SOC and total nitrogen (TN), respectively, and ergosterol were used as indicators to describe the impact of coverage type on soil fungi and microbial community (Anderson 2003; Joergensen and Emmerling 2006). The MBC:MBN, MBC:SOC and MBN:TN ratios were calculated from the MBC (chloroform-fumigation, TOC analysis), MBN (chloroform-fumigation, ninhydrin reaction, UV/VIS spectrometry), SOC (HCl fumigation, CHNS analysis), and TN (CHNS analysis) values published in Meyer et al. (2020, 2021a, b). The ergosterol determination was based on the method of Gong et al. (2001). Four grams of air-dried, milled soil were mixed with 12 mL methanol (1:3, w/v) and subsequently shaken for 60 min on a horizontal shaker (Kreisschüttler 3015, GFL, Burgwedel, Germany) and afterwards ultrasonically treated for 10 min (DT 514H, Bandelin electronics GmbH & Co. KG, Berlin, Germany). Then, the suspension was centrifuged for 10 min at 2000* g* (Universal 320, Hettich Lab Technology, Tuttlingen, Germany), and ultracentrifuged for 3 min at 7270* g* (Micro centaur, MSE Ltd, London, UK). Finally, 20 µL of the supernatant were analyzed with a high-performance liquid chromatography system with UV detection at 282 nm (HPLC 1200 series, Agilent technologies, Santa Clara, USA), equipped with a C_18_ LiChrospher® column (LiChrospher RP-18e, 5 µm, 100 Å, 250 × 4.6 mm, Merck KGaA, Darmstadt, Germany). For method validation, the soil was in advance spiked with ergosterol at concentrations of 0.5 and 5 mg kg^−1^ (*n* = 5 for each concentration). Recovery ranged between 96 ± 1 and 115 ± 2% and showed a relative standard deviation (RSD) of < 2%. Regression analysis of eight ergosterol standards in the range from 0.05 to 10 mg L^−1^ showed an excellent linear fit (*R*^2^ = 1) and a limit of detection (LOD) < 0.06 mg kg^−1^. For more detailed information on method validation see the supplementary information (SI Table 1 and SI Fig. 4).

### Analysis of mycotoxins in soil

Soil samples were analyzed for DON, NIV, and ZEN, based on a method by Mortensen et al. (2003), modified by Muñoz et al. (2017). Five grams of air-dried, milled soil (Planetary micro mill PULVERISETTE 7 premium line, Fritsch GmbH, Idar-Oberstein, Germany) were shaken with 15 mL methanol/water mixture (9:1, v/v) for 30 min on a horizontal shaker (Kreisschüttler 3015, GFL, Burgwedel, Germany) and subsequently treated for 10 min with ultrasonication (DT 514H, Bandelin electronics GmbH & Co.KG, Berlin, Germany). The suspension was 10 min centrifuged at 2000* g* (Universal 320, Hettich Lab Technology, Tuttlingen, Germany). An aliquot of 10 mL was subsequently evaporated to dryness under a nitrogen stream at 50 °C (Evaporatorsystem EVA-EC1-24-S, VLM Korrosions-Prüftechnik, Labortechnik & Dienstleistungen GmbH, Bielefeld, Germany), and the residues were reconstituted with 1 mL of mobile phase (methanol/water 1:1 v/v with 0.1% formic acid and 4 mM ammonia formiate). The solution was ultracentrifuged for 5 min at 7270* g* (Micro centaur, MSE Ltd, London, UK), and 20 µL of the supernatant were analyzed for mycotoxins with liquid chromatography–high-resolution mass spectrometer (LC-HRMS, Thermo Fisher Scientific, Waltham, USA), using a Hypersil GOLD™ C_18_ column (100 × 2.1 mm, 1.9 μm, Thermo Fisher Scientific, Waltham, USA). The mycotoxins were quantified with a matrix-matched calibration curve (1, 2.5, 5, 10, 25, 50, 75, and 100 µg L^−1^) prepared in soil extract (same procedure as for samples). All calibration standards for mycotoxins were purchased by Romer Labs Deutschland GmbH (Butzbach, Germany). Samples were considered positive when concentrations were above the lowest concentration level (LCL), which was 1 µg L^−1^ for DON, NIV, and ZEN, respectively, and corresponds to a soil concentration of 0.3 µg kg^−1^. For DON and NIV, ^13^C labelled internal standards were used as additional confirmation step. All mycotoxins were quantified in the negative ion mode, using the following mass-to-charge ratios: 356.1750 and 272.1701 for ^13^C-DON and ^13^C-NIV and 341.1240, 357.1195, and 317.1389 for the DON, NIV, and ZEN, respectively. For method validation, the soil was in advance spiked with DON, NIV, and ZEN at concentrations of 5, 15, and 50 µg kg^−1^. Recovery values ranged between 117 ± 11 and 144 ± 29% for DON, 119 ± 10 and 139 ± 22% for NIV, and 67 ± 7 and 126 ± 10% for ZEN. The RSD was ≤ 20% for all mycotoxins. Regression analyses of nine mycotoxin standards in the range from 0.5 to 100 µg L^−1^ showed a good linear fit (DON: *R*^2^ = 0.9725, NIV: *R*^2^ = 0.9888 and ZEN: *R*^2^ = 0.9937). For more detailed information on method validation see the supplementary information (SI Table 2, SI Figs. 5–7).

### Statistical analyses

Normality distribution of data was examined graphically with histograms and quantile–quantile plots. Mixed factorial ANOVAs with coverage time and soil depth as repeated factors and treatment as fixed factor were applied to determine significant differences between means. If significant interaction effects were occurring, additional ANOVAs, with least significance distance testing as post hoc test, were applied to locate significant differences. Variance homogeneity was confirmed with Levene’s test. Differences were reported as statistically significant if the probability of error was < 0.05. Method validation for ergosterol and mycotoxin analysis were based on ICH guideline Q2 (except LCL determination of mycotoxins). The LOD was calculated as 3.3σ/S, with σ as the standard deviation of the intercept of the regression line and S as the slope of the regression line calculated from the calibration standards (International Council for Harmonisation of Technical Requirements for Pharmaceuticals for Human Use 2005). For mycotoxin determination, the LCL was used as LOQ. The LCL determination based on the visual evaluation (empiric) methods, which has been suggested to provide more realistic values in complex matrices (Şengül 2016). According to this, the smallest standard of the matrix-matched calibration curve that gives a clear peak (signal-to-noise ratio > 10) was used to assess the LCL. For all results below the LCL, the LCL/2 was used for mean and standard deviation calculation (Ogden 2010). All statistical analysis was done with IBM SPSS Statistics 25.

## Results

### General soil properties under plastic and straw coverage

Soil temperature (Fig. 1a) was up to 2 °C (daily mean) or rather 6.5 °C (hourly) higher under PC than under SC, especially in spring (March–June) and in late-summer (August–October). The highest soil temperatures (hourly) reached 34.2 and 34.6 °C under PC in summer 2016 and 2019, whereas the highest soil temperatures under SC reached only 29.2 and 25.9 °C in the same periods (SI Table 4). In contrast, the soil moisture (Fig. 1b) was about 5–10 and 2–5% lower under PC compared to SC at the 5 and 35 cm soil depth, respectively, whereas the soil moisture at the 15 cm soil depth was partly higher under PC but the differences between treatments were generally small (mostly < 2%).

### The influence of plastic and straw coverage on microbial soil properties

**Fig. 2:**
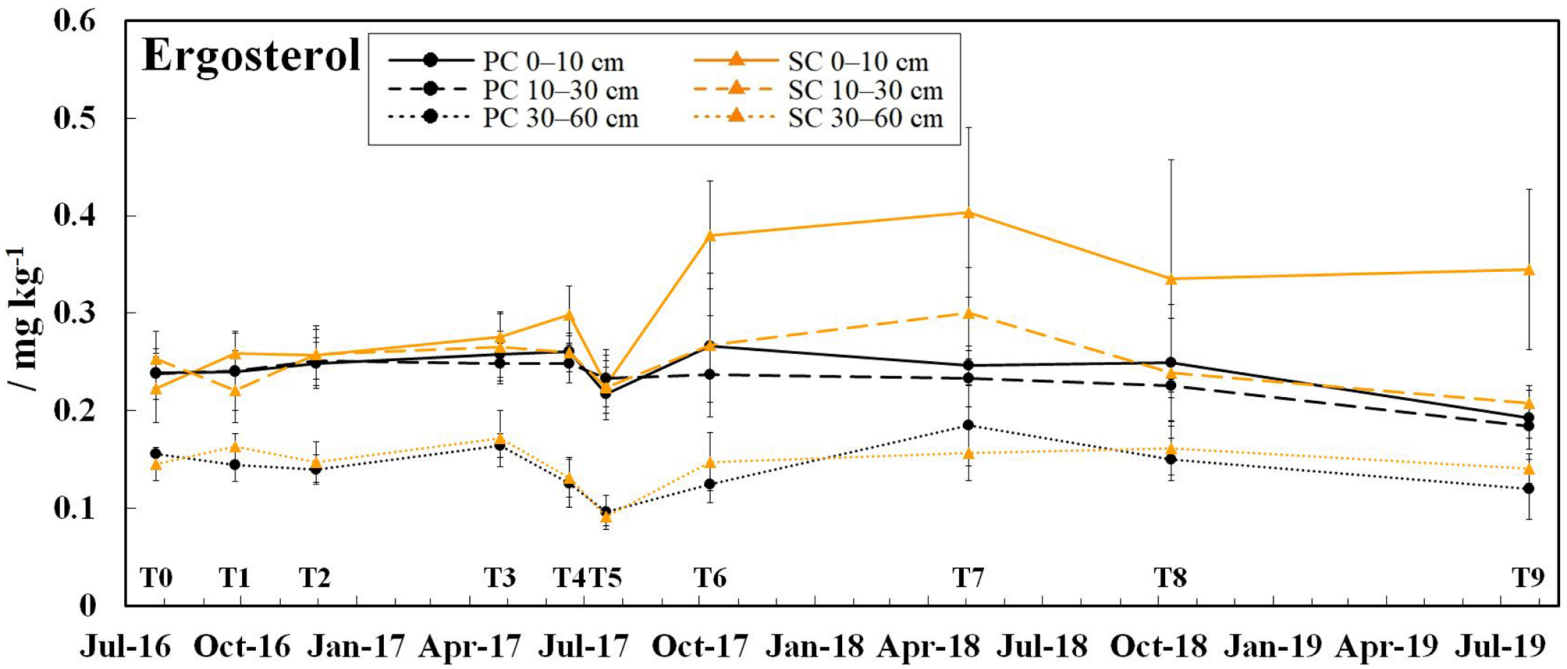
Ergosterol concentrations determined in the 0–10, 10–30, and 30–60 cm soil layer under plastic coverage (PC) and straw coverage (SC) at ten dates within the 3-year field experiment, respectively, shown as mean with standard deviation (*n* = 5)

**Fig. 3:**
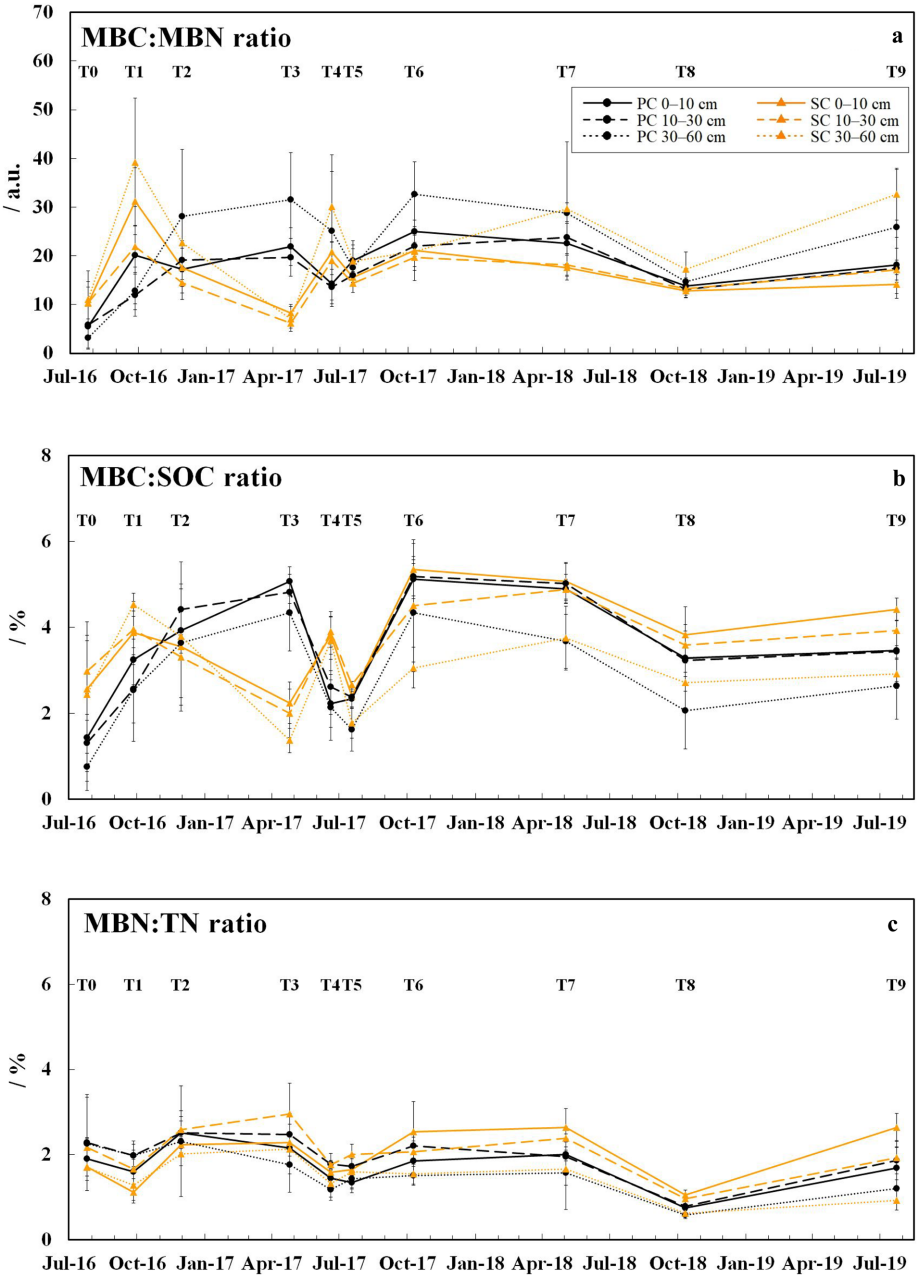
Elemental and eco-physiological ratios. **a** Microbial biomass carbon to nitrogen ratio (MBC:MBN ratio) determined in the 0–10, 10–30, and 30–60 cm soil layer under plastic coverage (PC) and straw coverage (SC) at ten dates within the 3-year field experiment, respectively, shown as mean with standard deviation (*n* = 5). **b** Microbial biomass carbon to soil organic carbon ratio (MBC:SOC ratio). **c** Microbial biomass nitrogen to total nitrogen ratio (MBN:TN ratio)

The ergosterol values (Fig. 2) ranged from 0.09 to 0.40 mg kg^−1^ and were by 0.09–0.16 mg kg^−1^ higher under SC compared to PC in the 0–10 cm soil layer from T6 to T9 (*p* ≤ 0.041). The lowest ergosterol values in the respective soil layers of both treatments (except 0–10 cm soil layer under SC at T9) were found at T5 (July 2017) and T9 (July 2019). The MBC:MBN ratios (Fig. 3a) were between 3 and 39 and showed wider ratios under PC than under SC at T2, T3 (all soil layers: *p* ≤ 0.004), T6 (30–60 cm soil layer: p = 0.013), and T7 (10–30 cm soil layer: *p* = 0.014). Conversely, narrower MBC:MBN ratios were observed under PC at T0 (30–60 cm soil layer: *p* = 0.034), T1 (all soil layers: *p* ≤ 0.021), and T4 (0–10 and 10–30 cm soil layer: *p* ≤ 0.019). The MBC:MBN ratios became wider in both treatments during the sampling period (*p* ≤ 0.038); however, the increase was stronger under PC (3.0–8.2 times) compared to SC (1.4–2.9 times). The MBC:SOC (Fig. 3b) and the MBN:TN ratios (Fig. 3c) were between 0.8–5.3 and 0.6–3.0%, respectively. The MBC:SOC ratios were higher under PC than under SC at T3 (all soil layers: *p* ≤ 0.001), whereas the opposite was observed at T0 (10–30 and 30–60 cm soil layer: *p* ≤ 0.048), T1 (10–30 and 30–60 cm soil layer: *p* ≤ 0.018), T4 (all soil layers: *p* ≤ 0.010), T8 and T9 (0–10 cm soil layer: *p* = 0.036). The MBN:TN ratios were by 0.3–1.0% higher under PC than under SC in the 0–10 cm soil layer from T5 to T9 (T5, T8 and T9: *p* ≤ 0.008).

### Mycotoxin occurrence in soil under plastic and straw coverage

The investigated mycotoxins DON, NIV and ZEN (Tables 2, 3, and 4) were detected in respectively 26.3, 8.3, and 8.7% of the samples and occurred primarily in the establishment period of the strawberries (T0–T2) and regarding DON also in the four months after fungicide application (T4–T6). The DON concentrations ranged from 0.3 (LCL) to 21.8 µg kg^−1^ and occurred mainly in the 0–10 and 10–30 cm soil layer at T1, T2, T4, T5, and T6. The DON concentrations were higher under SC than under PC in the 0–10 and 10–30 cm soil layer at T1 (6.4–7.7 vs. 1.4–1.6 µg kg^−1^) and at T5 (19.6–21.8 vs. 0.6–0.7 µg kg^−1^). DON was only sporadically detected in the 30–60 cm soil layer and at T0, T3, T7, T8, and T9. The NIV concentrations ranged from 0.3 (LCL) to 10.5 µg kg^−1^ and occurred primarily at T1 and T2. At T2, NIV showed a tendency to higher concentrations under PC compared to SC in all soil layers. ZEN was mainly detected at T0 and T2 in low concentrations (≤ 0.5 µg kg^−1^) and showed no differences between treatments.

**Table 2:** Deoxynivalenol (DON) concentrations determined in the 0–10, 10–30, and 30–60 cm soil layer under plastic coverage (PC) and straw coverage (SC) at ten dates in the 3-year field experiment, respectively. DON concentrations are shown as mean with standard deviation (*n* = 5). In brackets, the number of detects above the lowest concentration level (LCL) of 0.3 µg kg^−1^

**Treatment**	**Soil layer**	**Sampling**									
		T0	T1	T2	T3	T4	T5	T6	T7	T8	T9
		25.07.16	26.09.16	29.11.16	25.04.17	19.06.17	18.07.17	09.10.17	03.05.18	11.10.18	23.07.19
		DON	DON	DON	DON	DON	DON	DON	DON	DON	DON
	(cm)	(µg kg^−1^)	(µg kg^−1^)	(µg kg^−1^)	(µg kg^−1^)	(µg kg^−1^)	(µg kg^−1^)	(µg kg^−1^)	(µg kg^−1^)	(µg kg^−1^)	(µg kg^−1^)
PC	0–10 cm	< LCL	1.6 ± 3.1 (1)	3.4 ± 2.9 (4)	< LCL	1.1 ± 0.9 (3)	0.7 ± 1.3 (1)	0.9 ± 0.7 (3)	< LCL	< LCL	< LCL
	10–30 cm	< LCL	1.7 ± 3.5 (1)	3.5 ± 1.7 (5)	< LCL	1.7 ± 0.6 (5)	0.8 ± 0.9 (2)	1.7 ± 0.1 (5)	0.2 ± 0.2 (1)	< LCL	< LCL
	30–60 cm	< LCL	< LCL	< LCL	< LCL	0.6 ± 0.6 (2)	< LCL	0.5 ± 0.7 (1)	< LCL	< LCL	< LCL
SC	0–10 cm	1.9 ± 2.9 (2)	6.4 ± 3.2 (5)	3.8 ± 1.2 (5)	< LCL	0.9 ± 0.7 (3)	21.8 ± 15.0 (5)	1.1 ± 0.7 (4)	< LCL	< LCL	< LCL
	10–30 cm	< LCL	7.7 ± 2.3 (5)	0.5 ± 0.5 (2)	< LCL	1.1 ± 0.8 (3)	19.6 ± 17.5 (4)	0.5 ± 0.5 (2)	< LCL	0.3 ± 0.2 (1)	< LCL
	30–60 cm	< LCL	1.5 ± 1.8 (2)	< LCL	< LCL	0.7 ± 0.9 (2)	< LCL	< LCL	< LCL	< LCL	< LCL

< *LCL* all five samples below LCL

**Table 3:** Nivalenol (NIV) concentrations determined in the 0–10, 10–30, and 30–60 cm soil layer under plastic coverage (PC) and straw coverage (SC) at ten dates in the 3-year field experiment, respectively. NIV concentrations are shown as mean with standard deviation (*n* = 5). In brackets, the number of detects above the lowest concentration level (LCL) of 0.3 µg kg^−1^

**Treatment**	**Soil layer**	**Sampling**									
		T0	T1	T2	T3	T4	T5	T6	T7	T8	T9
		25.07.16	26.09.16	29.11.16	25.04.17	19.06.17	18.07.17	09.10.17	03.05.18	11.10.18	23.07.19
		NIV	NIV	NIV	NIV	NIV	NIV	NIV	NIV	NIV	NIV
	(cm)	(µg kg^−1^)	(µg kg^−1^)	(µg kg^−1^)	(µg kg^−1^)	(µg kg^−1^)	(µg kg^−1^)	(µg kg^−1^)	(µg kg^−1^)	(µg kg^−1^)	(µg kg^−1^)
PC	0–10 cm	< LCL	1.7 ± 3.5 (1)	7.9 ± 4.4 (4)	< LCL	< LCL	< LCL	< LCL	< LCL	< LCL	< LCL
	10–30 cm	< LCL	1.8 ± 3.6 (1)	10.5 ± 1.8 (5)	< LCL	< LCL	< LCL	< LCL	< LCL	< LCL	< LCL
	30–60 cm	< LCL	< LCL	3.7 ± 4.9 (2)	< LCL	< LCL	< LCL	< LCL	< LCL	< LCL	< LCL
SC	0–10 cm	< LCL	< LCL	5.5 ± 4.9 (3)	< LCL	< LCL	< LCL	< LCL	< LCL	0.2 ± 0.2 (1)	< LCL
	10–30 cm	< LCL	3.8 ± 5.1 (2)	7.6 ± 4.2 (4)	< LCL	< LCL	< LCL	< LCL	0.4 ± 0.5 (1)	< LCL	< LCL
	30–60 cm	< LCL	< LCL	< LCL	< LCL	< LCL	< LCL	< LCL	< LCL	0.2 ± 0.2 (1)	< LCL

< *LCL* all five samples below lowest concentration level

**Table 4:** Zearalenone (ZEN) concentrations determined in the 0–10, 10–30, and 30–60 cm soil layer under plastic coverage (PC) and straw coverage (SC) at ten dates in the 3-year field experiment, respectively. ZEN concentrations are shown as mean with standard deviation (*n* = 5). In brackets, the number of detects above the lowest concentration level (LCL) of 0.3 µg kg^−1^

**Treatment**	**Soil layer**	**Sampling**									
		T0	T1	T2	T3	T4	T5	T6	T7	T8	T9
		25.07.16	26.09.16	29.11.16	25.04.17	19.06.17	18.07.17	09.10.17	03.05.18	11.10.18	23.07.19
		ZEN	ZEN	ZEN	ZEN	ZEN	ZEN	ZEN	ZEN	ZEN	ZEN
	(cm)	(µg kg^−1^)	(µg kg^−1^)	(µg kg^−1^)	(µg kg^−1^)	(µg kg^−1^)	(µg kg^−1^)	(µg kg^−1^)	(µg kg^−1^)	(µg kg^−1^)	(µg kg^−1^)
PC	0–10 cm	< LCL	< LCL	0.2 ± 0.1 (2)	0.2 ± 0.1 (1)	< LCL	< LCL	< LCL	< LCL	< LCL	< LCL
	10–30 cm	0.2 ± 0.1 (1)	< LCL	0.4 ± 0.3 (3)	0.2 ± 0.1 (1)	< LCL	< LCL	< LCL	< LCL	< LCL	< LCL
	30–60 cm	< LCL	< LCL	0.2 ± 0.1 (2)	0.3 ± 0.2 (2)	< LCL	< LCL	< LCL	< LCL	< LCL	< LCL
SC	0–10 cm	0.5 ± 0.5 (2)	< LCL	0.3 ± 0.2 (2)	< LCL	< LCL	< LCL	< LCL	< LCL	< LCL	< LCL
	10–30 cm	0.6 ± 0.6 (2)	< LCL	0.2 ± 0.1 (2)	0.3 ± 0.4 (1)	< LCL	< LCL	< LCL	0.2 ± 0.1 (1)	< LCL	< LCL
	30–60 cm	0.4 ± 0.5 (1)	< LCL	0.3 ± 0.1 (3)	< LCL	< LCL	< LCL	< LCL	< LCL	< LCL	< LCL

< *LCL* all five samples below lowest concentration level

## Discussion

### How do the soil conditions under plastic and straw coverage influence soil fungi and microbial community?

The PC had no positive effect on fungal growth, despite of the elevated soil temperatures compared to SC (especially in the warmer season), which usually increases fungal growth (Pietikåinen et al. 2005; Bárcenas-Moreno et al. 2009). This is in contrast to former studies, which found an increased fungal biomass under plastic mulching (Subrahmaniyan et al. 2006; Muñoz et al. 2015). But as found that a temperature elevation of 4 °C for several years had no effect on microbial biomass, it was assumed that the temperature elevation under PC was possibly not large or long enough to affect fungal biomass. The larger fungal biomass in the topsoil (0–10 cm) under SC from T6 (October 2017) to T9 (July 2019) was attributed to the higher soil moisture and C-inputs under SC during that period (SI Fig. 2), which favor fungal growth (Dighton 2003; Swer et al. 2011). SOC particularly promote fungal growth if the biomass is fresh and have wide C:N ratios (Bossuyt et al. 2001) such as the applied wheat straw under SC, which usually have C:N ratios between 50 and 100 (Blume et al. 2016). Because the higher soil moisture under SC was already observed before October 2017 and also in the subsoil layer, the biomass entry might be the main driver for the increased fungal biomass. The low ergosterol values in July 2017 and July 2019 might reflect a reduced fungal biomass due to fungicide application in May/June (D’Mello et al. 1998; Magan et al. 2002).

The microbial community under PC showed, derived from MBC:MBN ratios, a stronger increasing and temporally higher fungal fraction (T3, T6, and T7) during the sampling period compared to SC. As fungal biomass has usually wider C:N ratios (≈ 10) than bacterial biomass (≈ 4) (Sylvia et al. 2005), wider MBC:MBN ratios indicate larger fungal fractions in microbial communities (Campbell et al. 1991). Because fungal biomass (ergosterol) under PC remained almost constant throughout the sampling period, the changed microbial community composition most likely results from a reduced bacterial fraction, which was possibly suppressed by drier soil condition and the mostly higher SOC under PC (SI Fig. 2; Allen et al. 1995; Bailey et al. 2002). The fungal biomass under SC was temporarily stronger reduced after fungicide treatments, because the soil under SC received larger fungicide loads due to the permeability of the straw cover to fungicides in contrast to the impermeable plastic covers (Meyer et al. 2021a). This in turn can temporally favor bacterial growth under SC (Martınez-Toledo et al. 1998; Monkiedje 2002) and hence may additionally shift microbial community composition. Generally, the high MBC:MBN ratios (> 20) during the sampling period point to a strong C entry into soil (Joergensen and Emmerling 2006), presumably due to root growth and exudation of the strawberry plants and aboveground biomass entry (only SC) (Meyer et al. 2021b).

The MBC:SOC and MBN:TN ratios are sensitive for changes in SOM (e.g., due to soil management or environmental changes) and can correlate positively with biomass inputs, soil C and N conversion to microbial biomass (microbial growth), and the degradability/availability of SOM for microorganism (Anderson 2003; Joergensen and Emmerling 2006). The larger MBC:SOC and MBN:TN ratios under SC from T6 (October 2017) to T9 (July 2019), primarily in the surface soil (0–10 cm), indicate a larger conversion of soil C and N resources to microbial biomass and hence a better available soil C and N for microorganism compared to PC (Anderson 2003; Joergensen and Emmerling 2006). This corresponds to the larger labile SOM fraction under SC (Meyer et al. 2021b), which is usually considered as fast-mineralizable and easily available substrate for microorganisms (Wander 2004), derived from the fresh biomass input under SC (Powlson et al. 1987; Meyer et al. 2021b).

### How do the soil conditions under plastic and straw coverage influence mycotoxin occurrence?

Solely the NIV concentrations at T2 support hypothesis 2 that the adaption of soil fungi to the modified microclimate under PC will trigger a higher mycotoxin occurrence. In contrast to hypothesis 2 and previous studies by Muñoz et al. (2015, 2017), the DON concentrations were particularly at T1 and T5 markedly higher under SC than under PC. The DON and NIV concentrations under PC (21.8 and 10.5 µg kg^−1^) were higher than those measured by Muñoz et al. (2017) in strawberry cultivation (3.0 and 1.1 µg kg^−1^). As the impervious plastic mulch impedes mycotoxin leaching from infested and contaminated plant materials into soil, the results provide a clear evidence that mycotoxins can be produced in situ in soil.

The results suggest that mycotoxin occurrence was stronger influenced by specific field treatment (fungicide application) and by the strawberry growth stage (establishment period) than by the mulching treatments. The biosynthesis of mycotoxins has been suggested as fungal adaptation to stress induced by a multitude of unfavorable growth conditions such as temperature and pH extremes, competition, water, and nutrient scarcity (Wheeler et al. 1991; Schmidt-Heydt et al. 2008; Reverberi et al. 2010; Venkatesh and Keller 2019) and the presence of fungicides (Magan et al. 2002). Thus, the mycotoxin occurrence in the strawberry establishment period (T0–T2) might be interpreted as fungal response to the interspecific competition between plants and microorganisms for available N-nutrients (Inselsbacher et al. 2010), induced by the strong plant (root) growth after strawberry plantation (Kumar and Dey 2011; Meyer et al. 2020). Particularly, the adaption of the microbial community to the crop change (Berg and Smalla 2009) induces changes and competition in fungal community (Garbeva et al. 2004; Qin et al. 2017), which can trigger fungi to mycotoxin production (Venkatesh and Keller 2019). This assumption is supported by the fact that mycotoxins were primarily detected in both upper soil layers (0–10 and 10–30 cm), representing the root zone of strawberries. The DON occurrence from T4 to T6 was interpreted as stress response to the fungicide applications (Magan et al. 2002), which was stronger under SC because of the larger fungicide loads received compared to PC (Meyer et al. 2021a). Beside competition and fungicide effects, soil parameters such as temperature, moisture and pH are known to influence *Fusarium* growth and mycotoxin biosynthesis in soil (Marin et al. 1995; Sweeney and Dobson 1998; Ramirez et al. 2006; Schmidt-Heydt et al. 2008). Soil pH remained neutral (6.5–7.9) during sampling period (SI Fig. 1; Meyer et al. 2021b) and hence had no effect on fungal growth and on mycotoxin biosynthesis (Marin et al. 1995; Sweeney and Dobson 1998). But the temperature maxima and minima for *Fusarium* growth (above 31–37 °C or below 5 °C) were respectively exceeded in topsoil under PC in summer 2016 and 2019 and in all soil layers of both treatments in each winter 2016–2019 (SI Table 4). As DON and NIV biosynthesis is mainly regulated by temperature (Llorens et al. 2004), the cold temperatures in winter might enhanced mycotoxin production and hence mycotoxins occurrence at T2.

Beside factors influencing mycotoxin production in soil, additionally, the fate of mycotoxins must be considered when evaluating mycotoxin concentrations in soil. Literature about the fate of DON, NIV, and ZEN in soil is very limited, but some indications were found for microbial degradation and leaching into running waters (Elmholt 2008; Schenzel et al. 2012b; Kolpin et al. 2014). DON, NIV, and ZEN are stable against transformation by abiotic parameters such as temperature, pH, and hydrolysis (Lauren and Smith 2001; Pitt et al. 2012). The DON and NIV concentrations increased in both mulching treatments during establishment period of strawberries (T0–T2), which implies that mycotoxin production exceeds mycotoxin reduction due to degradation and leaching processes, leading to an accumulation of NIV and DON. No indications were found that the chemically stable and water-soluble DON and NIV (Table 1) were relocated into deeper soil layers during sampling period, which would indicate leaching. In general, leaching under PC seems unlikely because of the impeded rainfall infiltration and the partially occurring lateral and ascending water flows (Ruidisch et al. 2013; Meyer et al. 2021b). Nevertheless, the DON and NIV concentrations at T2 and T5 vanished (almost) completely to the next sampling, which was presumably induced by microbial degradation (Elmholt 2008) because the aforementioned abiotic transformation and leaching can be excluded. If plant uptake of the water-soluble DON and NIV can occur and hence be a possible fate pathway was not yet investigated.

To the best of our knowledge, this was the first study which investigated the influence of plastic mulching on fungi and mycotoxins in soil in the temporal course of a multiannual plant cultivation. Clear evidence was shown that mycotoxins can be biosynthesized in situ in soil. Regarding the investigated mulching treatments, plastic mulching had no clear positive effects on soil fungi and mycotoxin occurrence compared to the more traditional straw mulching, presumably because its effects on soil temperature, moisture, pH, and SOM were mostly small under the present humid, temperate climate (Meyer et al. 2020, 2021a, b). However, as usually stronger effects on soil conditions were reported under warmer and more arid climates (Gan et al. 2013; Steinmetz et al. 2016), further studies under different climate conditions are necessary to better assess the influence of plastic mulching on mycotoxin occurrence. As mycotoxin occurrence in soil was influenced by plant growth stage and fungicide application, further research is required with different crops and fungicides to gain a better understanding of their impact on mycotoxin biosynthesis.

## Supplementary Information

Below is the link to the electronic supplementary material.

Supplementary file1 (DOCX 1339 KB)

## Data Availability

The datasets used and/or analyzed during the current study are available from https://doi.org/10.6084/m9.figshare.14447127.v2.

## Abbreviations

DON: Deoxynivalenol
NIV: Nivalenol
ZEN: Zearalenone
SOM: Soil organic matter
PC: Plastic-covered ridge-furrow system with subsurface drip irrigation
SC: Straw-covered ridge-furrow system with subsurface drip irrigation
SOC: Soil organic carbon
TN: Total nitrogen
C:N ratio: Carbon-to-nitrogen ratio
MBC: Microbial biomass carbon
MBN: Microbial biomass nitrogen
LOD: Limit of detection
RSD: Relative standard deviation
LCL: Lowest calibration level
