# Supplementary material for: How does multiannual plastic mulching in strawberry cultivation influence soil fungi and mycotoxin occurrence in soil?
Source: Mycotoxin Res. 2022 Mar 22;38(2):93–105. doi: 10.1007/s12550-022-00451-5 (PMC9038900; doi:10.1007/s12550-022-00451-5)
Supplement: Supplementary file 1 — Supplementary file1 (DOCX 1339 KB) [file 12550_2022_451_MOESM1_ESM.docx]

**Supplementary Information**

**SI Figure 1** Soil pH (in 0.01 M CaCl_2_) determined in the 0–10, 10–30 and 30–60 cm soil layer under plastic coverage (PC) and straw coverage (SC) at ten dates in the three-year field study, shown as mean with standard deviation (n=5). Data summarized from Meyer et al. (2020; 2021a, b)


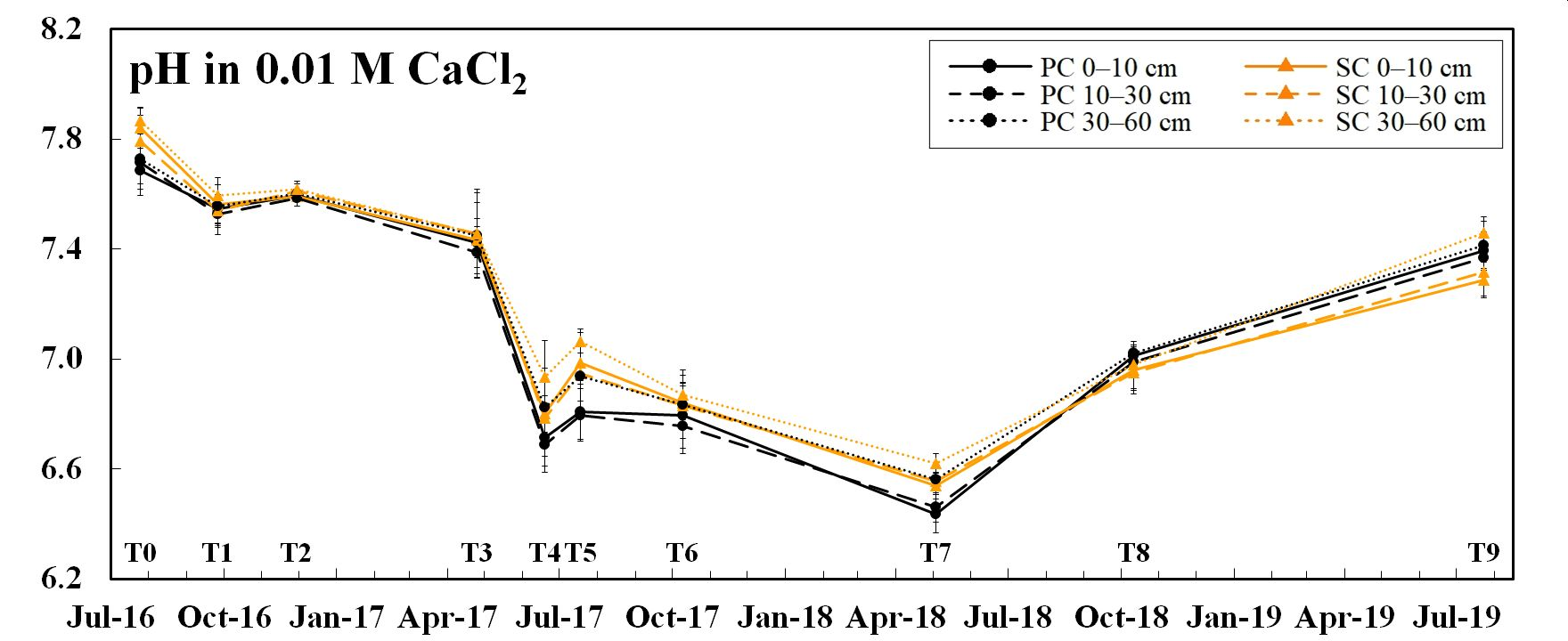


**SI Figure 2** Soil organic carbon (SOC) determined in the 0–10, 10–30 and 30–60 cm soil layer under plastic coverage (PC) and straw coverage (SC) at ten dates in the three-year field study, shown as mean with standard deviation (n=5). Data summarized from Meyer et al. (2020; 2021a, b)


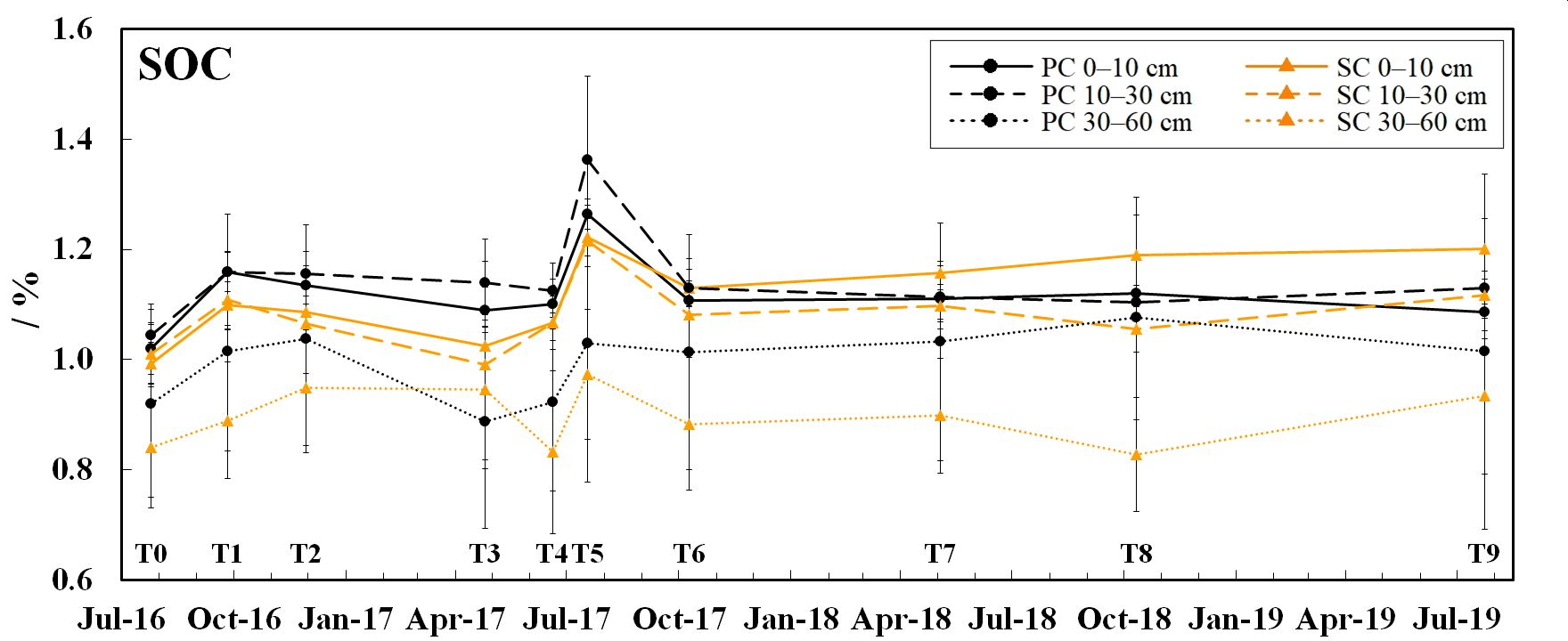


**SI Figure 3** Carbon to nitrogen ratio (C:N ratio) determined in the 0–10, 10–30 and 30–60 cm soil layer under plastic coverage (PC) and straw coverage (SC) at ten dates in the three-year field experiment, shown as mean with standard deviation (n=5). Data summarized from Meyer et al. (2020; 2021a, b)


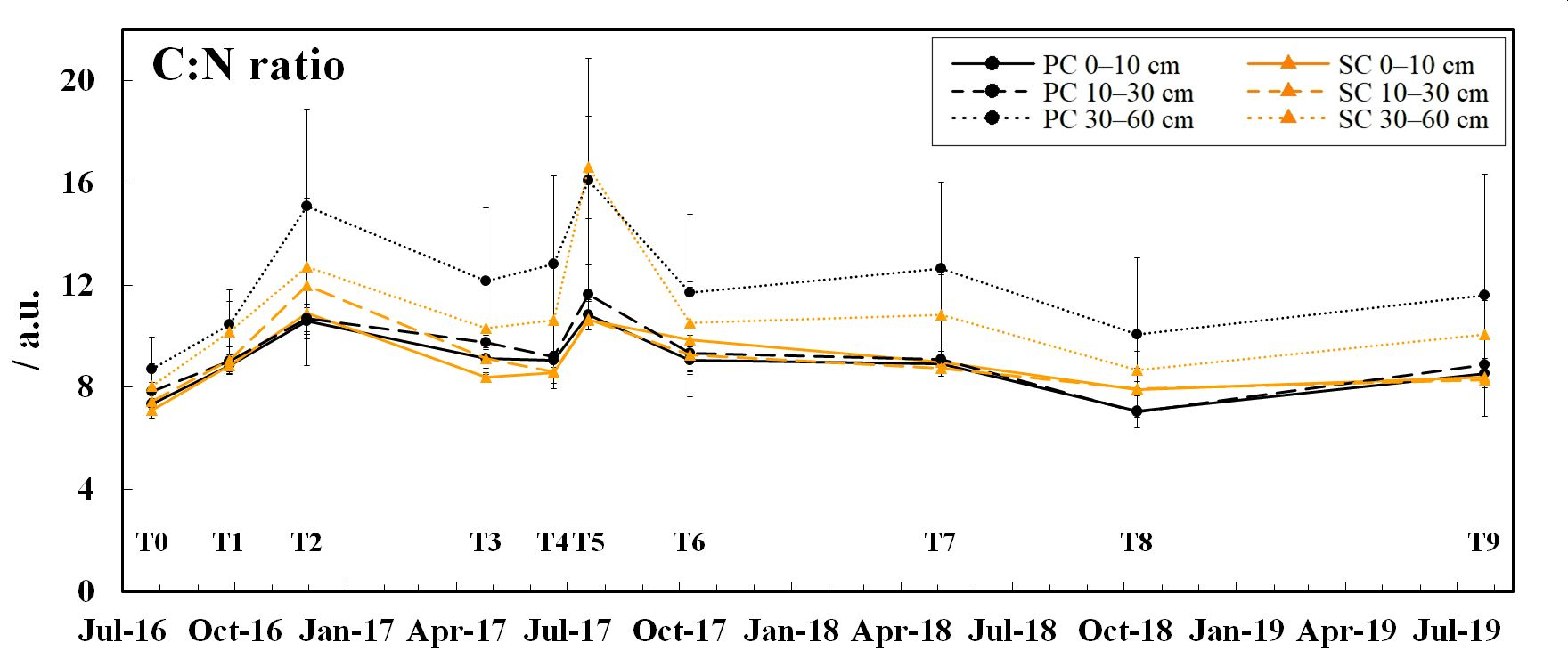


**Validation data of ergosterol method**

For recovery determination air-dried, milled soil from the sampling site was used. Soil was spiked with ergosterol for two nominal concentrations (0.5 and 5 mg kg^-1^). A control was used to correct the spiked samples by the field concentration. Ergosterol extraction was conducted as described in the Materials and Methods section and extracts were analyzed with HPLC-UV. Ergosterol concentrations were calculated from a calibration curve, comprising eight ergosterol standards in methanol (0.05, 0.1, 0.2, 0.5, 1, 2, 5 and 10 mg L^-1^).

**SI Table 1** Determination of ergosterol recovery from soil spiked at two nominal concentrations of 0.5 and 5 mg kg^-1^. Soil extracts were analyzed with HPLC-UV. Values are given as means with standard deviation and relative standard deviation (RSD)

| Sample | Replicates | Concentration soil extract | Soil concentration (control-corrected) | Recovery | RSD |
| --- | --- | --- | --- | --- | --- |
|  |  | / mg L^-1^ | / mg kg^-1^ | / % | / % |
| Control | 5 | 0.089±0.007 |  |  |  |
| 0.5 mg kg^-1^ | 5 | 0.280±0.003 | 0.574±0.010 | 114.7±2.1 | 0.9 |
| 5 mg kg^-1^ | 5 | 1.691±0.014 | 4.806±0.041 | 96.1±0.8 | 1.8 |

**SI Figure 4** Ergosterol calibration curve in methanol


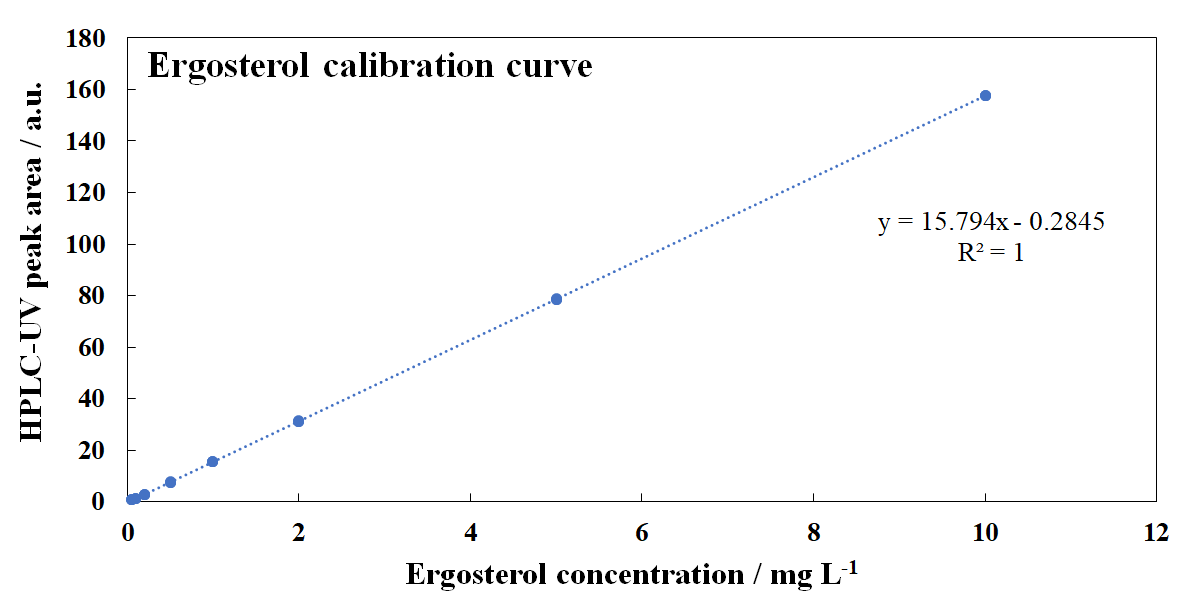


**Validation data of mycotoxin method**

For recovery determination air-dried, milled soil from the sampling site was used. Soil was spiked with a mycotoxin mix to three nominal concentrations (5, 15 and 50 µg kg^-1^). Mycotoxin extraction was conducted as described in the Materials and Methods section and extracts were analyzed with LC-HRMS. Mycotoxin concentrations were calculated with a matrix-matched calibration curve (prepared in soil extract, gained with the same extraction procedure as described for mycotoxin analysis). The calibration curves comprised nine mycotoxin concentrations (0.5, 1, 2.5, 5, 10, 25, 50, 75 and 100 µg L^-1^).

**SI Table 3** Recovery of the mycotoxins deoxynivalenol, nivalenol and zearalenone from soil spiked with three nominal concentrations of 5, 15 and 50 µg kg^-1^. Values are given as means with standard deviation and relative standard deviation (RSD)

| Sample | Replicates | Deoxynivalenol | |  | Nivalenol | |  | Zearalenone | |
| --- | --- | --- | --- | --- | --- | --- | --- | --- | --- |
|  |  | Recovery | RSD |  | Recovery | RSD |  | Recovery | RSD |
|  |  | / % | / % |  | / % | / % |  | / % | / % |
| 5 µg kg^-1^ | 5 | 130±7 | 5.4 |  | 119±10 | 8.4 |  | 67±7 | 10.4 |
| 15 µg kg^-1^ | 3 | 117±11 | 9.4 |  | 119±24 | 20.2 |  | 82±4 | 4.9 |
| 50 µg kg^-1^ | 5 | 144±29 | 20.1 |  | 139±22 | 15.8 |  | 126±10 | 7.9 |

**SI Figure 5** Matrix‑matched calibration curve of deoxynivalenol


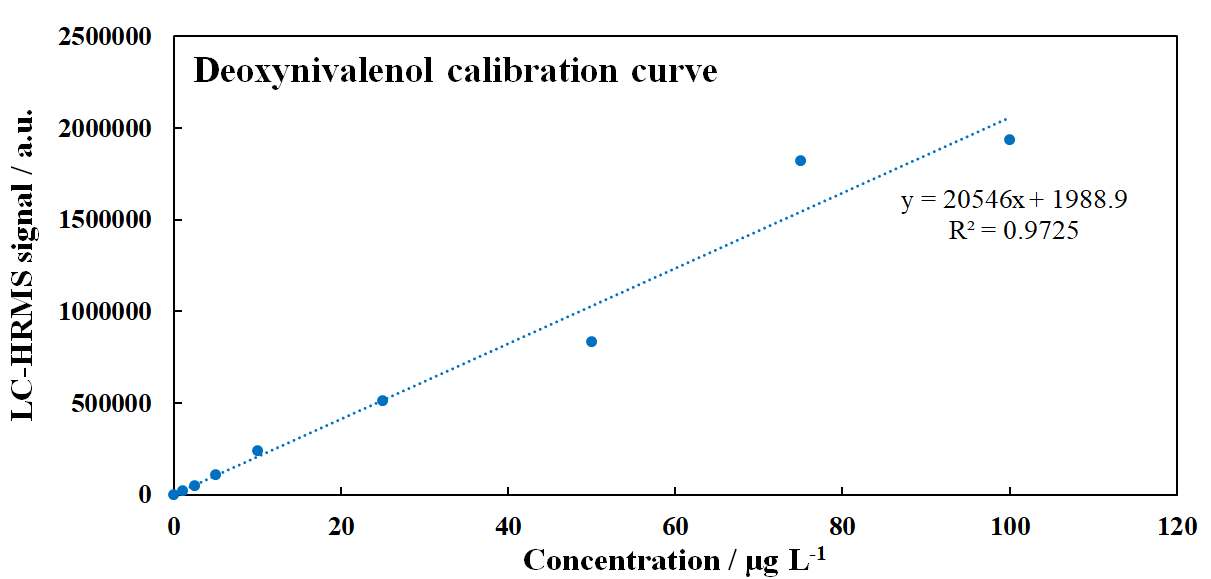


**SI Figure 6** Matrix‑matched calibration curve of nivalenol


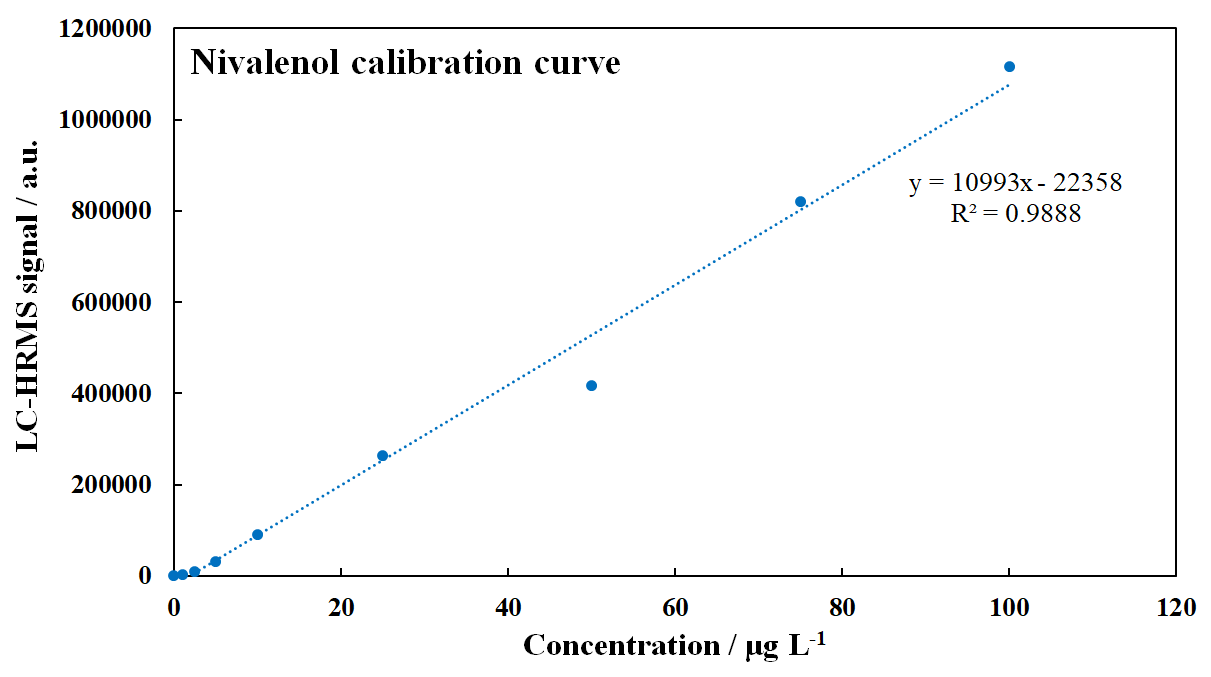


**SI Figure 7** Matrix‑matched calibration curve of zearalenone


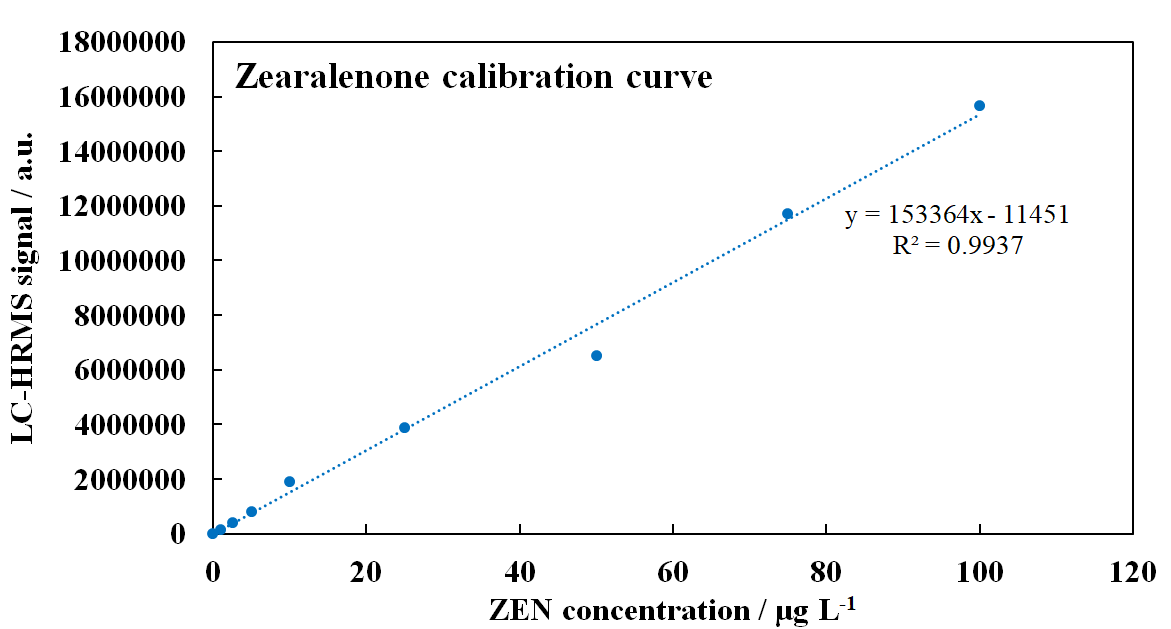


**SI Table 4** The temperature range of the 100 largest and lowest soil temperatures measured under plastic and straw coverage at 5 cm soil depth in the three‑year field study

| Sampling year | Soil layer | Plastic coverage | |  | Straw coverage | |
| --- | --- | --- | --- | --- | --- | --- |
|  |  | Maximum temperature | Minimum temperature |  | Maximum temperature | Minimum temperature |
|  | / cm | / °C | / °C |  | / °C | / °C |
| 2016 | 0–10 cm | 28.3 – 34.2 | 0.1 – 0.9 |  | 25.1 – 29.2 | 0.2 – 0.6 |
|  | 10–30 cm | 25.9 – 28.9 | 1.0 – 1.7 |  | 24.2 – 26.7 | 0.9 – 2.2 |
|  | 30–60 cm | 24.2 – 25.6 | 2.3 – 3.1 |  | 23.5 – 24.4 | 1.4 – 2.9 |
|  |  |  |  |  |  |  |
| 2017 | 0–10 cm | 22.6 – 24.7 | -1.7 – -0.7 |  | 21.3 – 22.4 | -2.2 – -1.1 |
|  | 10–30 cm | 21.3 – 22.4 | -0.5 – -0.3 |  | 20.5 – 21.3 | -0.9 – -0.5 |
|  | 30–60 cm | 20.3 – 20.7 | 0.5 – 0.6 |  | 19.7 – 20.1 | 0.2 – 0.4 |
|  |  |  |  |  |  |  |
| 2018 | 0–10 cm | 24.2 – 29.5 | -0.8 – -0.2 |  | 21.7 – 23.9 | -1.6 – -0.5 |
|  | 10–30 cm | 21.9 – 22.8 | -0.2 – 0.0 |  | 21.3 – 22.4 | -0.4 – -0.2 |
|  | 30–60 cm | 21.7 – 23.0 | 0.9 – 1.1 |  | 20.5 – 21.1 | 0.7 – 0.9 |
|  |  |  |  |  |  |  |
| 2019 | 0–10 cm | 27.7 – 34.6 | -0.2 – 0.0 |  | 21.7 – 25.9 | -0.2 – -0.1 |
|  | 10–30 cm | 25.4 – 30.1 | 0.4 – 0.6 |  | 20.9 – 22.4 | 0.3 – 0.4 |
|  | 30–60 cm | 23.7 – 25.6 | 1.7 – 2.1 |  | 19.5 – 20.3 | 1.6 – 1.9 |
